# Dosimetric Comparison of Organs At Risk Between Artificial Intelligence-Based Auto-Contouring and Manual Contouring for High-Risk Prostate Cancer Radiotherapy: A Retrospective Study

**DOI:** 10.7759/cureus.91810

**Published:** 2025-09-08

**Authors:** Masumi Kawaguchi, Shinji Sugahara, Masataka Hoshina, Masaya Noguchi, Masato Takanashi, Kouichi Masuda, Yoshiaki Katada, Kazuhiro Saito

**Affiliations:** 1 Department of Radiology and Radiation Oncology, Tokyo Medical University Ibaraki Medical Center, Inashiki-gun, JPN; 2 Department of Radiology, Tokyo Medical University Ibaraki Medical Center, Inashiki-gun, JPN; 3 Department of Radiology, Tokyo Medical University, Tokyo, JPN

**Keywords:** artificial intelligence, auto-contouring, deep learning, dose-volume histogram, high-risk prostate cancer, intensity-modulated radiotherapy, non-inferiority study, radiation treatment planning, volumetric modulated arc therapy

## Abstract

Background and aim

This study compared the dose volume histogram (DVH) parameters of the organs at risk (OARs) between an artificial intelligence (AI)-based auto-contouring method and conventional manually drawn contours in radiation treatment planning for high-risk prostate cancer.

Methods

Fifteen treatment plans for high-risk prostate cancer treated with 76 Gy in 38 fractions using volumetric modulated arc therapy (VMAT) were retrospectively analyzed. For each patient, both manual contouring (MC) by an experienced radiation oncologist and automated contouring (AC) by OncoStudio (Oncosoft, Seoul, Republic of Korea) were performed, and then the DVH parameters of the OARs were compared between MC and AC. Primary outcome measures were dose constraints (V20 to V75) for the rectum, bladder, and the femoral heads. T-test statistical analysis was performed.

Results

The analysis showed no statistically significant differences between the MC and AC groups in all the DVH parameters for OARs (p>0.05). The mean difference in rectum V50 was 1.8% (95%CI: -0.87 to 4.47%, p=0.178), and the mean difference in bladder V70 was 1.6% (95%CI: -0.12 to 3.32%, p=0.067). The effect sizes R² ranged from 0.001 to 0.115, and the observed differences were small and clinically acceptable.

Conclusion

In high-risk prostate cancer radiotherapy planning, AC with OncoStudio demonstrated non-inferiority in the OARs dose assessment compared to MC. There were no statistically significant differences in the DVH parameters of all the OARs, and the observed differences were clinically acceptable. The introduction of AC is expected to streamline the OARs contouring process while maintaining clinical equivalence.

## Introduction

In radiation therapy planning, accurate contouring of organs at risk (OARs) is a crucial step in reducing adverse events and ensuring treatment success [1-3]. In particular, accurate contouring is required for high-risk prostate cancer radiation therapy because doses delivered to adjacent OARs, such as the rectum and bladder, directly affect the incidence of post-treatment adverse events and quality of life [4,5]. Traditionally, contouring of these OARs has been manually performed by radiation oncologists or medical physicists, but this task is time-consuming and labor-intensive, and has been reported to contribute to inter- and intra-observer variability [6-8].

The rapid development of artificial intelligence (AI) technology has led to significant progress in automatic segmentation technology in medical image processing [9], with AI applications transforming various aspects of radiation oncology practice [10]. In particular, the development of auto-contouring (AC) software based on deep learning technology, including convolutional neural networks for target segmentation, is expected to contribute greatly to the efficiency and standardization of radiation therapy planning [11,12]. Several previous studies have reported the geometric accuracy of AC systems in prostate cancer radiotherapy, showing that the Dice similarity coefficient for OARs is generally 0.80 or higher, and in some cases 0.90 or higher [13,14]. In addition, Kawula et al. reported that the dose volume histogram (DVH) differences for the bladder and rectum were within clinically acceptable levels [15]. Furthermore, a study by Arjmandi et al. showed that AC can reduce manual work time from approximately 25 minutes to less than one minute [16]. Duan et al. evaluated a commercial software, INTContour, and reported no significant dose differences between automatic and manual contouring (MC) in most OARs [17]. Nevertheless, comprehensive evaluation of the clinical efficacy of AI-based AC remains to be conducted, including a detailed analysis based on DVH parameters. We have been testing a commercial software, OncoStudio (Oncosoft, Seoul, Republic of Korea); however, there were few publications that evaluated DVH parameters.

In prostate cancer, the balance between local control by high-dose irradiation and dose sparing to adjacent OARs is important, and a more detailed understanding of the impact of accurate contouring of OARs on the quality of treatment plans is necessary. In this study, we conducted a comprehensive DVH study of OncoStudio's AC for OARs, including the rectum, bladder, and bilateral femoral heads in the treatment planning for high-risk prostate cancer.

## Materials and methods

Patient selection

This study was a single-center retrospective study and was approved by the Ethics Committee of Tokyo Medical University (approval number: T2025-0017). Fifteen patients were enrolled in the study and underwent definitive volumetric modulated arc therapy (VMAT) between January 2023 and March 2025 for high-risk localized prostate cancer. Eligibility criteria were as follows: (1) histologically confirmed prostate cancer, (2) completed definitive VMAT at 76 Gy in 38 fractions, (3) treatment planning CT available, and (4) diagnostic images of sufficient image quality available. Exclusion criteria were reirradiated cases and cases with image artifacts.

Image acquisition and delineation of the OARs

All patients underwent treatment planning CT scans in the supine position with the bladder filled. CT scans were performed with a 1 mm slice thickness. The rectum, bladder, and both femoral heads were evaluated as OARs in radiotherapy for prostate cancer. For each patient, two contouring methods were performed.

Manual Contouring (MC)

A radiation oncologist with over 30 years of experience manually contoured the OARs, including the rectum, bladder, and both femoral heads, on the treatment planning system (TPS) according to the standard guidelines of the institution, which was used as the gold standard. The rectum was contoured to include five slices above and below the planning target volume (PTV) of the prostate, the entire volume of the bladder, and the entire femoral heads on both sides.

Auto-Contouring (AC)

AC was performed using OncoStudio version 2.0 on the same patient CT images. OncoStudio employs a deep learning model using a convolutional neural network, enabling automatic segmentation of risk organs around the prostate. It uses a model that is specifically trained to identify OARs in the pelvic region and is particularly accurate in delineating the contours of the rectum and bladder.

When there was a discrepancy in the slice level of the structures between MC and AC by OncoStudio, adjustments were made so that both were located on the same slice plane. Specifically, for each structure (rectum, bladder, and both femoral heads), the slice planes of MC and AC were aligned at the top and bottom ends, and any discrepancies were manually removed so that the slice levels matched fully. This adjustment eliminated the effect of discrepancies in slice levels in the comparison of DVH parameters, thereby allowing more accurate evaluation.

Treatment planning and OARs assessment index

The treatment plans were evaluated using the following procedure. First, the CT image data of 15 prostate cancer patients at our hospital was processed using the OncoStudio AC software. The generated contour data (structures) and CT data were loaded into the Monaco 6.2 TPS (Elekta, Stockholm, Sweden) via a USB memory in DICOM format, and then transferred to the Pinnacle3 TPS (Philips, Cleveland, USA) used in our clinical practice.

After adjusting the slice position as described above for both MC and AC structure sets in Pinnacle3, the same treatment planning algorithm was applied to both contouring sets to create standard VMAT plans with a prescription dose of 76 Gy in 38 fractions and the following dose constraints: for the rectum: V70<5%, V60<10%, V50<20%, V40<30%; for the bladder, V70<10%, V60<20%, V40<40%; and for the femoral heads, V25=0%.

The primary endpoints were compared for the following DVH parameters for OARs: V75, V70, V50 for rectum and bladder; and V30 and V20 for bilateral femoral heads. These DVH parameters are standard evaluation indices in prostate cancer radiotherapy and are widely adopted in international clinical trial protocols such as the Radiation Therapy Oncology Group (RTOG) [5]. In particular, rectum V70 and V50 are associated with adverse events such as late rectal bleeding, and bladder V70 is associated with cystitis symptoms. These DVH parameters were extracted directly from the Pinnacle3 TPS and used for statistical analysis.

Statistical analysis

Statistical analysis was performed using JMP Pro 16.0 (JMP Statistical Discovery LLC, North Carolina, USA). Paired t-tests were performed to detect differences between DVH parameters in the MC and AC groups. Mean differences, 95% confidence intervals (CI), and effect sizes (R²) were calculated for each comparison. In this study, the effect sizes R² are classified as follows: negligible effect if R² <0.01, small effect if 0.01≤ R² <0.06, medium effect if 0.06≤ R² <0.14, and large effect if R² ≥0.14.

Given the small sample size (n=15), the criteria for clinical non-inferiority were predefined as follows: The disparity in each DVH parameter between AC and MC falls within the clinical tolerance limits for the rectum and bladder, with ΔV70 <5%, ΔV50 <10%, and for the femoral heads, with ΔV30 <5%. The symbol "Δ" denotes the subtraction of DVH parameters from MC to AC.

For all statistical analyses, a corrected p value of less than 0.05 was considered statistically significant. Given the exploratory nature of this study, effect size and clinical relevance were also important in interpreting the results.

## Results

Patient characteristics

The characteristics of the 15 patients are shown in Table 1.

**Table 1:** Patient characteristics PSA: Prostate Specific Antigen

Number of patients	15
Age (years): median and range	79 (61-86)
PSA (ng/mL): median and range	8.34 (3.63-70.88)
Gleason score	
3+4	3 cases (20.0%)
4+3	2 cases (13.3%)
4+4	7 cases (46.7%)
4+5	2 cases (13.3%)
5+5	1 case (6.7%)
Clinical T classification	
T2a	8 cases (53.3%)
T2c	5 cases (33.3%)
T3a	1 case (6.7%)
T3b	1 case (6.7%)

The median age was 79 years (range: 61-86 years), and the clinical stage was T2a-T3b. The median prostate specific antigen (PSA) level was 8.34 ng/mL (range: 3.63-70.88 ng/mL). The Gleason scores ranged from 3+4 to 5+5, with 4+4 being the most common in seven patients (46.7%), followed by 3+4 in three patients (20.0 %), 4+3 in two patients (13.3%), 4+5 in two patients (13.3%), and 5+5 in one patient (6.7%). The TNM classification was T2a in eight patients (53.3%), T2c in five patients (33.3%), T3a in one patient (6.7%), and T3b in one patient (6.7%). All patients met the National Comprehensive Cancer Network (NCCN) guidelines' high-risk classification [18] and completed VMAT in 76 Gy in 38 fractions.

Statistical comparison of DVH parameters of all OARs between MC and AC

The DVH parameters of the OARs between the MC and the AC groups are compared in Table 2.

**Table 2:** Comparison of auto-contouring and manual contouring for the DVH parameters for OARs OARs: organs at risk; CI: confidence interval; DVH: Dose-volume histogram; V75, V70, V50, V30, V20: percentage volume of organ receiving ≥ 75, 70, 50, 30, and 20 Gy, respectively.

Structure	Parameter	Manual contouring (Mean ± SD, %)	Auto-contouring (Mean ± SD, %)	Mean difference (%)	95% CI	Effect size (R²)	p
Rectum	V75	0.00±0.00	0.00±0.00	0	---	---	---
	V70	1.87±1.20	1.53±1.31	0.33	-0.64 to 1.31	0.017	0.489
	V50	16.0±3.29	14.2±3.60	1.8	-0.87 to 4.47	0.064	0.178
Bladder	V75	1.80±1.42	1.27±1.39	0.53	-0.56 to 1.62	0.035	0.324
	V70	9.67±1.96	8.07±2.46	1.6	-0.12 to 3.32	0.115	0.067
	V50	23.9±3.97	22.3±4.73	1.6	-1.78 to 4.98	0.032	0.341
Femoral head R	V30	1.33±3.05	0.73±1.24	0.6	-1.20 to 2.40	0.016	0.5
	V20	20.7±15.9	19.6±12.9	1.07	-10.12 to 12.25	0.001	0.847
Femoral head L	V30	3.13±4.21	1.73±2.64	1.4	-1.32 to 4.12	0.038	0.301
	V20	33.7±18.7	28.5±15.6	5.13	-8.22 to 18.5	0.022	0.438

Data are presented as mean ± standard deviation for the 15 patients. Effect sizes are presented as R² values from response screening analysis. P-values were calculated using paired t-tests.

In the rectal dose constraint assessment, a discrepancy of 0.33% was identified between the MC group (1.87±1.20%) and the AC group (1.53±1.31%) in V70, which was deemed statistical insignificant (p=0.489). In the rectal V50, a 1.8% discrepancy was observed between the MC group (16.0±3.29%) and the AC group (14.2±3.60%), which did not reach statistical significance (p=0.178). The effect sizes were very small (R² <0.07), which meant that practical differences could be ignored.

For bladder V75, a 0.53% difference was observed between the MC group (1.8±1.42%) and the AC group (1.27±1.39%), which was statistically insignificant (p=0.324, R² =0.035). For bladder V70, a 1.6% difference was observed between the MC group (9.67±1.96%) and the AC group (8.07±2.46%), which was also statistically insignificant (p=0.067, R² =0.115). For bladder V50, the difference between the two groups was 1.6%, indicating statistical insignificance (p=0.341, R² =0.032).

For the right femoral head V30, a difference of 0.6% was observed between the MC group (1.33±3.05%) and the AC group (0.73±1.24%), showing statistical insignificance (p=0.500, R² =0.016). For the right femoral head V20, the difference between the two groups was 1.07%, and the effect size was very small (p=0.847, R² =0.001). For the left femoral head, differences of 1.4% at V30 and 5.13% at V20 were observed, but neither reached statistical significance (p=0.301 and p=0.438, respectively).

**Figure 1:**
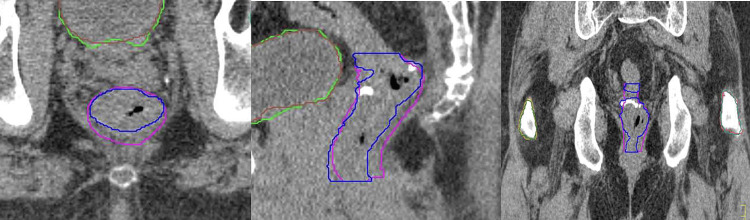
A comparison of the rectum MC (blue) and AC (pink) results on multiple planes The image also includes brown (MC) and green (AC) bladder contouring; MC: Manual contouring; AC: Auto-contouring.

**Figure 2:**
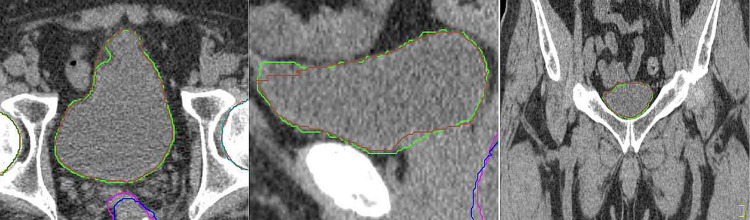
A comparison of the bladder MC (brown) and AC (green) results on multiple planes The image also includes MC (blue) and AC (pink) rectum contouring; MC: Manual contouring; AC: Auto-contouring.

**Figure 3:**
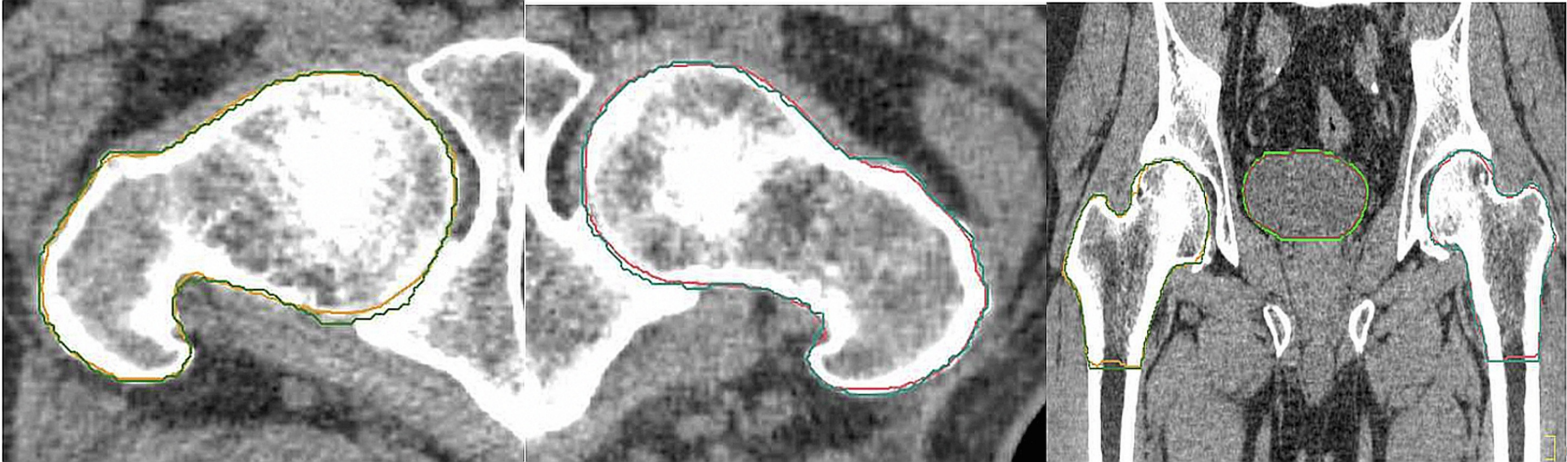
Comparisons of the right femoral head MC (green) and AC (orange), left femoral head MC (light blue) and AC (red) on multiple planes MC: Manual contouring; AC: Auto-contouring.

Figures 1-3 show comparisons of contouring for the rectum, bladder, and bilateral femoral heads in the same case across multiple planes, with MC in blue (rectum), brown (bladder), green (right femoral head), and light blue (left femoral head), and AC in pink (rectum), green (bladder), orange (right femoral head), and red (left femoral head).

OncoStudio accurately recognized anatomical boundaries and showed high agreement with MC, properly depicting the 3D organ shapes including the anterior walls of the rectum, the entire bladder volume from the anal canal to the rectosigmoid junction, and the complete cortical bone boundaries of both femoral heads.

Forest plot analysis for the differences of the mean DVH parameters between MC and AC

**Figure 4:**
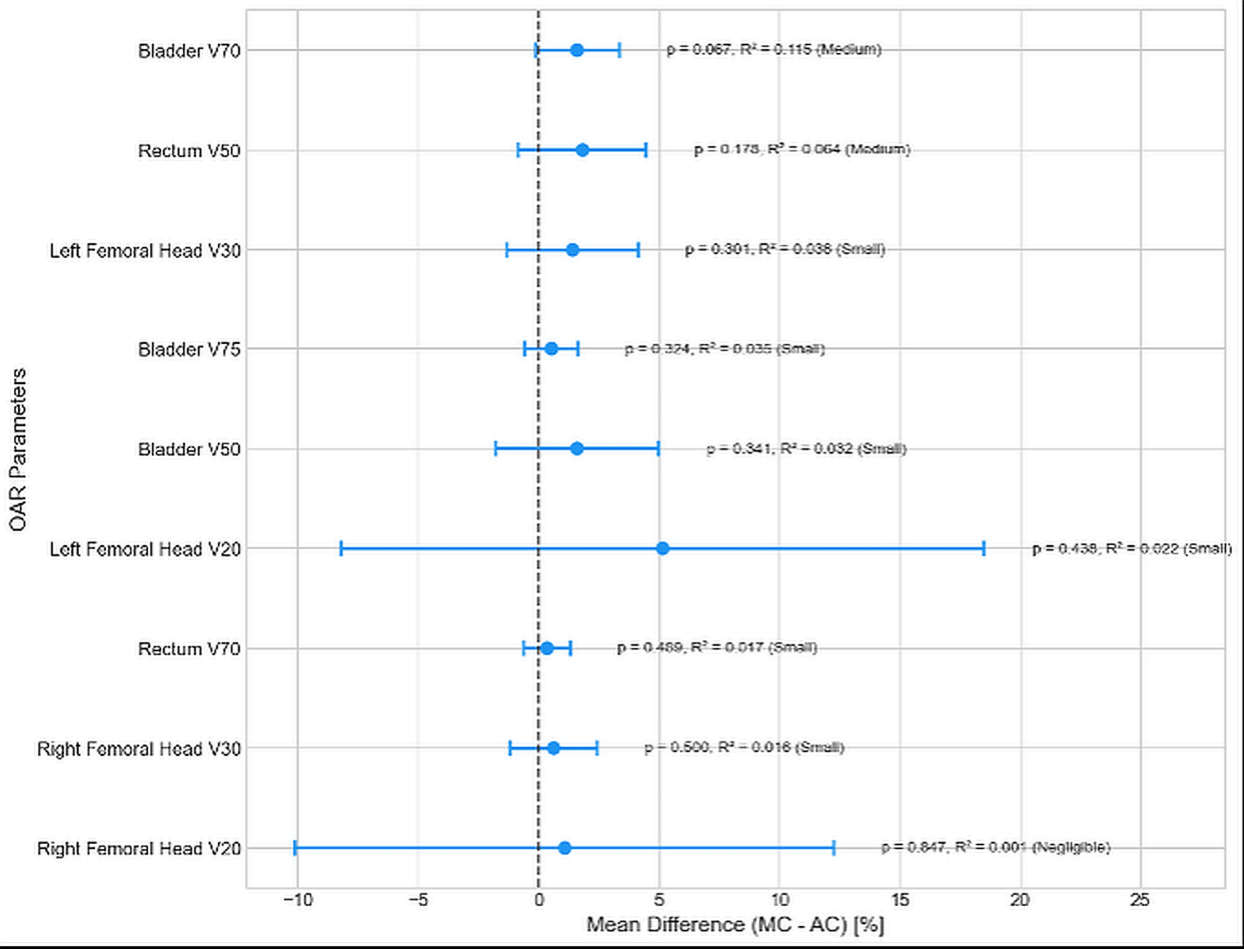
Forest plot of the differences of mean DVH parameters between MC and AC for each OAR in the descending order of effect size (R²), with each 95% confidence interval. OAR: organ at risk; MC: manual contouring; AC: auto-contouring; V75, V70, V50, V30, V20: percentage volume of organ receiving ≥ 75, 70, 50, 30, and 20 Gy, respectively.

Figure 4 presents the forest plots of the differences of mean DVH parameters between MC and AC for each OAR, arranged in the descending order of effect size (R²), along with each 95% CI indicated as a blue error bar.

Bladder V70 (R² =0.115) showed the largest effect size among all DVH parameters we studied, but the difference was not statistically significant (p=0.067). No statistically significant differences were found between MC and AC for any DVH parameter (p>0.05). From this plot, it can also be confirmed that each of the confidence intervals for all DVH parameters includes a value of 0, indicating that there were no statistically significant differences between the MC and AC groups.

Boxplot comparisons of DVH parameters for OARs between MC and AC

**Figure 5:**
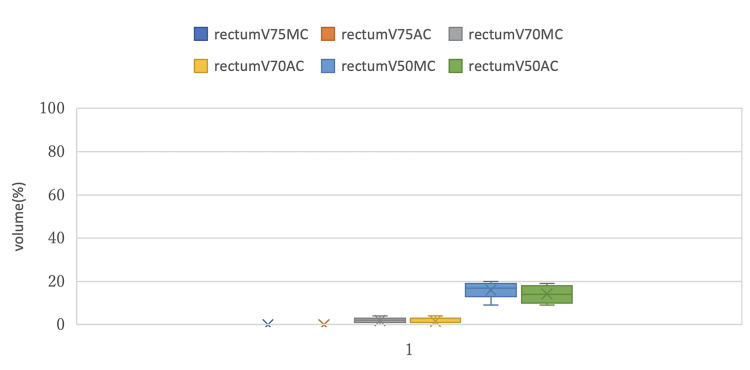
Comparison of the box plots of DVH parameters for the rectum between MC and AC DVH: Dose-volume histogram; MC: manual contouring; AC: auto-contouring.

**Figure 6:**
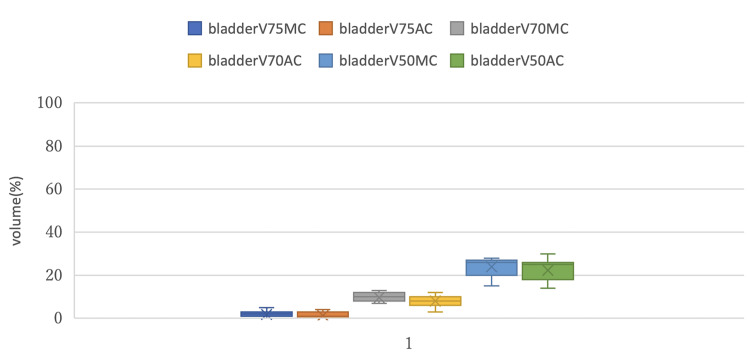
Comparison of the box plots of DVH parameters for the bladder between MC and AC DVH: Dose-volume histogram; MC: manual contouring; AC: auto-contouring.

**Figure 7:**
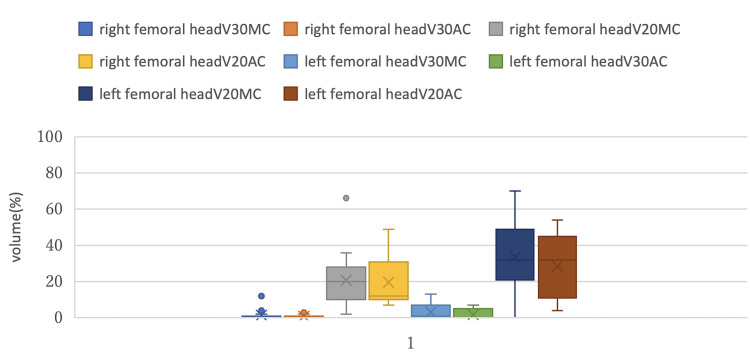
Comparison of box plots of the DVH parameters for the femoral heads between MC and AC DVH: Dose-volume histogram; MC: manual contouring; AC: auto-contouring.

Figures 5-7 show box plots that compare DVH parameters of each OAR between MC and AC.

Box plots are used to visually grasp the distribution of data, with the center line of the box representing the median, the upper and lower ends of the box representing the third quartile (75th percentile) and the first quartile (25th percentile), respectively, and the tips of the whiskers representing the maximum and minimum values.

Figure 5 compares the box plots of rectum V75, V70 and V50 between the MC and AC groups. The rectum V75 was 0% in each group. The rectum V50 tended to be slightly higher in the MC group, but no statistically significant differences were obtained as described earlier.

Figure 6 compares the bladder V75, V70 and V50 between the MC and AC groups. There was some separation in the V70 distribution between the two groups. This is consistent with the fact that bladder V70 showed the largest effect size (R² =0.115) among all DVH parameters and showed a trend toward statistical significance (p=0.067).

In Figure 7, comparisons of the femoral head DVH parameters (V30, V20) were made between the MC and AC groups. For the right femoral head V20, the box plots showed larger variations, but the effect size was the smallest (R² =0.001), and the difference was not statistically significant between MC and AC. For left femoral head V20, additional variations were identified; however, the mean V20 difference between MC and AC was not statistically significant as described earlier. In other words, these differences were within the clinically acceptable range.

DVH comparisons between MC and AC

**Figure 8:**
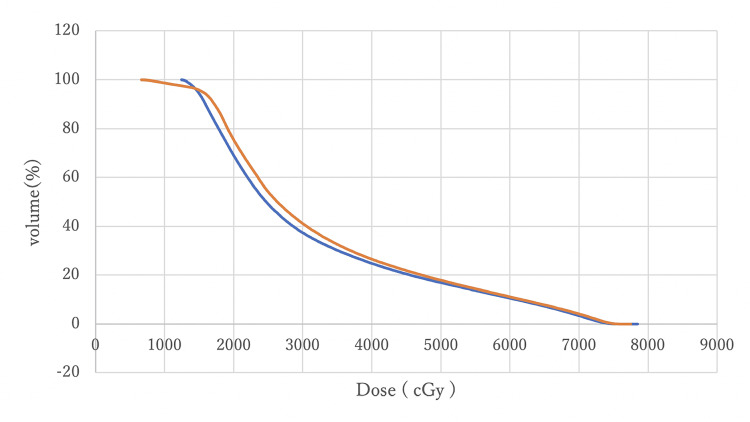
A comparison of DVH curves for the rectum between MC (blue) and AC (orange) DVH: Dose-volume histogram; MC: manual contouring; AC: auto-contouring.

**Figure 9:**
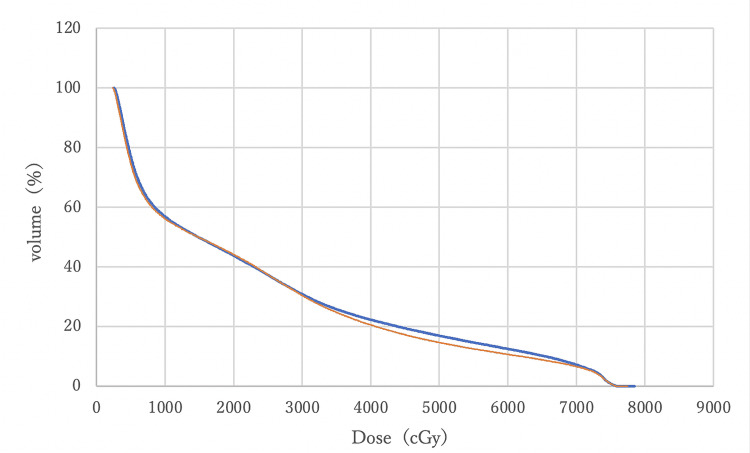
A comparison of DVH curves for the bladder between MC (blue) and AC (orange) DVH: Dose-volume histogram; MC: manual contouring; AC: auto-contouring.

**Figure 10:**
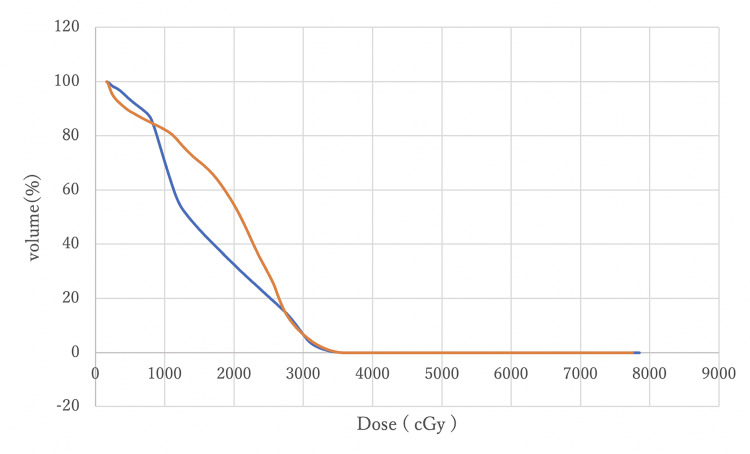
A comparison of DVH curves for the left femoral head between MC (blue) and AC (orange) DVH: Dose-volume histogram; MC: manual contouring; AC: auto-contouring.

**Figure 11:**
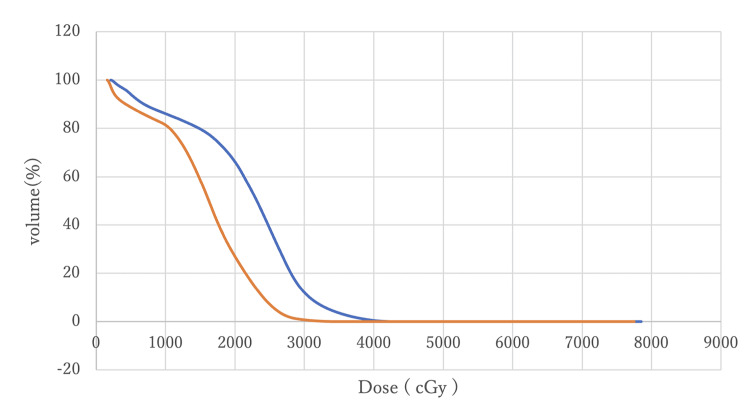
A comparison of DVH curves for the right femoral head between MC (blue) and AC (orange) DVH: Dose-volume histogram; MC: manual contouring; AC: auto-contouring.

Figures 8-11 compare DVH curves between MC and AC for each OAR for a particular patient.

Figure 8 indicates that DVHs for the rectum MC and AC were almost overlapping, with minimal differences at V70 and V50. This overlap suggests that AC provides equivalent rectal protection as MC. Figure 9 shows that bladder DVHs have some differences between MC and AC at higher doses, but these differences were considered clinically negligible. In addition, the shapes of the curves were similar, suggesting that the risk of late bladder toxicity may be comparable. Although some differences were observed between the MC and AC curves on both sides of the femoral heads at doses between 20 Gy and 30 Gy, no statistically significant differences were observed, and the differences were within the clinically acceptable range. These visual evidences support the statistical analysis and demonstrate that AC with OncoStudio produces clinically equivalent dose distributions as MC.

Clinical non-inferiority assessment

Based on the predefined clinical non-inferiority criteria, the AC group showed non-inferiority to the MC group in DVH parameters for all the OARs. The differences in rectum V70 and V50 (0.33% and 1.8%, respectively), bladder V70 and V50 (1.6% and 1.6%, respectively), and femoral head V30 (right: 0.6%, left: 1.4%) were all within clinical tolerance. These small differences were considered clinically negligible.

## Discussion

We evaluated the clinical performance of an AI-based AC software, OncoStudio, for OARs in radiotherapy planning for high-risk prostate cancer. Comparison of the DVH parameters of the OARs revealed that AC was non-inferior to MC. The results of this study indicate that AC with OncoStudio is clinically equivalent to MC across all dose constraints for OARs in high-risk prostate cancer radiotherapy. In particular, both contouring methods fully met the constraints of rectum V70<20%, bladder V70<35% and femoral heads V50<5% recommended by the Quantitative Analysis of Normal Tissue Effects in the Clinic (QUANTEC) guideline and RTOG [19,20], and there appeared to be no clinically important differences between the two methods with regards to the risk of late adverse events. Our results also showed no statistically significant differences in doses to the OARs. Effect sizes were generally small (R² ranging from 0.001 to 0.115), and observed differences were within clinically acceptable limits.

Several studies have reported on the clinical usefulness of AC for OARs. Cha et al. reported that automatic segmentation based on deep learning showed a high concordance rate with MC in prostate cancer cases, and reduced contouring time by an average of 66% [21]. However, most of these studies focused on anatomical concordance rates, and the evaluation of the DVH parameters, which are the most clinically important indices, was limited.

Our study focused on OARs in high-risk prostate cancer cases and evaluated the impact of AC on DVH parameters, which are the most clinically important indices. From a clinical perspective, it is known that the doses to major OARs, such as the rectum and bladder, are directly associated with the incidence of late adverse events. Once again, both contouring methods fully met the constraints based on the QUANTEC and RTOG guidelines, thereby maintaining clinically safe treatment plans [19,20].

In a recent study, Kawula et al. performed a dose evaluation of deep learning-based AC in prostate VMAT and reported that the DVH difference between the bladder and rectum was within a clinically acceptable range [15]. The results of the present study are consistent with these previous studies and support the clinical usefulness of AC in the specific patient population of high-risk prostate cancer.

Among all the DVH parameters analyzed, bladder V70 demonstrated the most substantial difference between the two contouring methods. This parameter showed the largest effect size (R² =0.115) and approached statistical significance (p=0.067), with AC consistently yielding lower values than MC (8.07% vs 9.67%). This systematic difference suggests that the two contouring approaches may differ in their delineation of bladder boundaries, particularly in regions exposed to higher radiation doses.

The clinical significance of this finding warrants consideration, as V70 represents a critical dosimetric parameter associated with late bladder toxicity. While both contouring methods remained within established safety guidelines, the consistent directional difference observed suggests potential systematic variations in how AC and MC define bladder contours in high-dose regions.

The mechanisms underlying this difference remain unclear from our current analysis. Potential contributing factors may include variations in bladder volume estimation, differences in boundary recognition algorithms, or systematic variations in how each method handles anatomical uncertainties. A comprehensive understanding of these differences would require additional analyses including total bladder volume comparisons, spatial overlap assessments using geometric indices such as the Dice coefficient, and detailed evaluation of contour boundaries in relation to dose distribution patterns.

Our study has several limitations. First, the sample size was small (n=15), which reduced the statistical power to detect minor variations. Second, as this was a single-center retrospective study, the results should be generalized with caution. Third, the potential for selection bias cannot be excluded given the small number of cases (n=15) selected over a multi-year period (January 2023 to March 2025). The criteria for case selection and the representativeness of the selected cases within the broader population of high-risk prostate cancer patients treated at our institution during this period were not systematically evaluated. Fourth, our evaluation was constrained to DVH parameters and did not compare the anatomical accuracy of contouring or the actual contouring time. These limitations may affect the generalizability of our findings. Future studies should include validation in larger, multicenter, prospective studies, quantitative evaluation of contouring time, and detailed analysis of anatomical accuracy.

## Conclusions

In radiotherapy planning for high-risk prostate cancer, AC with OncoStudio demonstrated comparable DVHs for OARs when compared to MC. No statistically significant differences were observed in the DVH parameters of all OARs, and the observed differences were within clinically acceptable limits. Specifically, the differences in rectum V70 and V50, bladder V70 and V50, and bilateral femoral head V30 were 0.33% and 1.8%, 1.6% and 1.6%, and 0.6% and 1.4%, respectively, all of which met the predefined clinical non-inferiority criteria. Furthermore, both contouring methods fully satisfied the dose constraints recommended by QUANTEC and RTOG guidelines, indicating no clinically important differences regarding the risk of late adverse events.

These results suggest that the implementation of AC using OncoStudio is expected to enhance the efficiency of the treatment planning process while preserving clinical equivalence. The potential to significantly reduce the time burden required for conventional MC while maintaining treatment safety and quality was demonstrated. However, this study was conducted at a single institution with a small number of cases, and validation through larger multicenter prospective studies is necessary. Furthermore, through continuous improvement of AC technology and the establishment of appropriate implementation protocols for clinical practice, contributions to standardization and efficiency enhancement of radiation treatment planning are anticipated.
